# Comparative characterization of Crimean-Congo hemorrhagic fever virus cell culture systems with application to propagation and titration methods

**DOI:** 10.1186/s12985-023-02089-w

**Published:** 2023-06-19

**Authors:** Hongzhao Li, Greg Smith, Melissa Goolia, Peter Marszal, Bradley S. Pickering

**Affiliations:** 1 National Centre for Foreign Animal Disease, Canadian Food Inspection Agency, Winnipeg, MB Canada; 2 Department of Medical Microbiology and Infectious Diseases, College of Medicine, Faculty of Health Sciences, University of Manitoba, Winnipeg, MB Canada; 3 Department of Veterinary Microbiology and Preventive Medicine, College of Veterinary Medicine, Iowa State University, Ames, IA USA

**Keywords:** Crimean-Congo hemorrhagic fever virus, Cell line, Cell culture, Titration, SW-13, Vero E6, HuH-7, BSR-T7/5, Plaque assay, TCID50

## Abstract

Crimean-Congo hemorrhagic fever orthonairovirus (CCHFV) is a biosafety level 4 and World Health Organization top priority pathogen. Infection leads to an often fatal hemorrhagic fever disease in humans. The tick-borne virus is endemic in countries across Asia, Europe and Africa, with signs of spreading into new regions. Despite the severity of disease and the potential of CCHFV geographic expansion to cause widespread outbreaks, no approved vaccine or treatment is currently available. Critical for basic research and the development of diagnostics or medical countermeasures, CCHFV viral stocks are commonly produced in Vero E6 and SW-13 cell lines. While a variety of in-house methods are being used across different laboratories, there has been no clear, specific consensus on a standard, optimal system for CCHFV growth and titration. In this study, we perform a systematic, side-by-side characterization of Vero E6 and SW-13 cell lines concerning the replication kinetics of CCHFV under different culture conditions. SW-13 cells are typically cultured in a CO_2_-free condition (SW-13 CO_2_^−^) according to the American Type Culture Collection. However, we identify a CO_2_-compatible culture condition (SW-13 CO_2_^+^) that demonstrates the highest viral load (RNA concentration) and titer (infectious virus concentration) in the culture supernatants, in comparison to SW-13 CO_2_^−^ and Vero E6 cultures. This optimal viral propagation system also leads to the development of two titration methods: an immunostaining-based plaque assay using a commercial CCHFV antibody and a colorimetric readout, and an antibody staining-free, cytopathic effect-based median tissue culture infectious dose assay using a simple excel calculator. These are anticipated to serve as a basis for a reproducible, standardized and user-friendly platform for CCHFV propagation and titration.

**Supplementary Information:**

The online version contains supplementary material available at 10.1186/s12985-023-02089-w.

## Background

CCHFV is a biosafety level 4 pathogen, and has been listed by World Health Organization as a top priority pathogen expected to result in severe future outbreaks [1, 2]. Although CCHFV infections in many cases result in a mild, nonspecific febrile illness, some patients develop a lethal hemorrhagic disease [3]. Fatality rate ranges from 5 to 30%, but up to 80% case fatality has been reported in some outbreaks [3–6]. Currently there are no approved vaccines or specific therapeutics against the disease, and medical treatments rely mostly on supportive care.

CCHFV belongs to the genus *Orthonairovirus*, family *Nairoviridae*, order *Bunyvirales*, according to the taxonomy update in 2019 [7]. It is an enveloped virus with a negative-sense, tripartite RNA genome including the small (S), medium (M) and large (L) segments, which encode the nucleoprotein, glycoprotein precursor and RNA-dependent RNA polymerase, respectively [3]. The virus is maintained and transmitted in tick-vertebrate host-tick cycles [3]. Among the at least 30 species of Ixodid (hard-body) ticks found to carry the virus, those from the genus *Hyalomma* are the most predominant reservoir and vector. Wild and domestic animals can carry infected ticks and serve as viral amplification hosts following tick bites. However, with the exceptions of immunodeficient, newborn or humanized mice and cynomolgus macaques as known susceptible hosts, animals do not show prominent signs of illness with CCHFV infection. This poses great risk to people in contact with infected hosts, as human infections occur through tick bites and exposure to body fluid or tissues containing the virus [8]. In addition, nosocomial infection has also been reported [9].

CCHFV is the most geographically widespread pathogen among tick-borne viruses and the second most geographically widespread pathogen among arboviruses (after Dengue virus). Its distribution involves Asia (from Western China to the Middle East and Turkey), Europe (Eastern and Southeastern countries) and Africa (majority of the continent). The vast geographic range reflects that CCHFV hosts tolerate broadly diverse environments [3, 8]. Signs are also emerging that CCHFV may expand outside traditional endemic regions, as cases have been reported in new areas such as western Europe. Such expansion may be due to the emergence of favorable ecological environment for viral hosts due to climate change, introduction of infected ticks by migratory birds or introduction of infected animals by livestock trade [3, 6, 8, 10–12].

CCHFV viral stocks are produced in the laboratory for use in research and development of diagnostics, vaccines and therapeutics. The virus can be isolated from the brain of experimentally infected suckling mice [13, 14], or grown in cell cultures [4, 13, 14]. In comparison, cell culture-based propagation methods, with the elimination of animal use and the ease of less technical complexity, are more frequently utilized. A number of cell lines were found to be permissive to CCHFV replication [4, 15]. Among these, Vero cell lines, primarily Vero E6 [4, 13, 14, 16–27], and SW-13 [8, 17–21, 24, 26, 28–41] appear to be the most frequently used in CCHFV culture experiments. However, significant variations and a lack of specific details can be found across methods of in-house derived culture conditions in different laboratories. There has been no clear, specific consensus on an optimal culture condition for high yield and rapid CCHFV harvest. For representative examples, in one study the entire viral propagation method was described as “CCHFV (strain IbAr10200) was propagated in Vero E6 cells”, with no specific details or reference to the literature [42]. A similar case was found, where the viral stock was amplified in HuH-7 cells [17]. In another study, the methods of viral propagation in SW-13 cells and plaque assay titration methods were referred to a Master’s thesis, which was not found by our search on the internet [43]. Furthermore, cell culture supernatants [44–46] and cell pellet lysates [13, 14, 47] were used to generate viral stocks in different studies. However, no data have been available comparing the yield or quality of stock production from the two portions of viral culture. Concerning high yield and rapid CCHFV harvest, few studies have provided time course data to identify timing of peak viral titers. Flick et al. (2003) collected supernatants of SW-13 cells on day 7 post-infection (7 DPI) with CCHFV IbAr10200 as viral stocks [43]. However, in our study viral titers peaked on 2 and 3 DPIs for CCHFV IbAr10200 and Kosovo Hoti strains, respectively, in SW-13 cells, and dropped sharply following the peak DPIs (see below). A similar early peak titer was shown by Dai et al. (2021) for CCHFV Asian strain YL16070 on 1 DPI in SW-13 cells [15].

In addition, CCHFV titration is largely limited to immunostaining methods, often using an in-house CCHFV antibody available from the respective individual laboratories [14, 16, 28, 48], which hinders reproducibility and standardization. In the context of an indirect immunofluorescence assay [14, 16, 28, 48], as often used in CCHFV quantification, the demand for a fluorescence imaging resource is not able to be met by a simple light microscope. Altogether, these variations paired with uncertainties and limitations may make it confusing for researchers new to the field in deciding what materials and methods to start with, while in general posing a challenge for maintaining reproducibility across different laboratories.

In this study, we conducted a comprehensive review of the literature on major cell lines used for CCHFV propagation and titration, a summary of the findings illustrating the most commonly used cell lines, Vero E6 and SW-13. We then tested replication characteristics of CCHFV in different culture conditions, with side-by-side comparison between Vero E6 and SW-13. We identified an optimal culture condition for CCHFV propagation, and subsequently developed new titration methods: a plaque assay based on immunostaining using a standard, commercially available CCHFV antibody and a colorimetric readout; and a cytopathic effect (CPE)-based median tissue culture infectious dose (TCID50) assay, which does not need antibody staining and uses a simple, user-friendly excel calculator. These findings are anticipated to be useful for establishing CCHFV propagation and titration methods characterized by availability of standard reagents, reproducibility and user-friendliness.

## Methods

### Literature search for cell lines used in CCHFV culture

A PubMed search was performed covering all-time literature. We used the key word “Crimean-Congo hemorrhagic fever” in the PubMed search, which returned 1,917 results. Since this number is practically manageable for a subsequent manual screening we did not use any additional filter in the search in order to maximize review coverage. We were able to quickly read through the titles and abstracts on PubMed using the following display options: Format = Abstract, Sort by = Most recent and Per page = 200, and select original experimental studies that involved the use of CCHFV, which we then examined for information on CCHFV culture and titration.

### CCHFV and host cell lines

The standard CCHFV IbAr10200 strain (GenBank accession numbers NC_005302.1, MH483988.1 and AY947891.1 for the S, M and L segments, respectively; passage 4) was a gift from Public Health Agency of Canada [8, 49, 50]. IbAr10200 is the default strain used throughout the study unless specified otherwise. CCHFV Kosovo Hoti (GenBank accession numbers DQ133507, EU037902 and EU044832 for the S, M and L segments, respectively; passage 7) was purchased from European Virus Archive—Global (https://www.european-virus-archive.com/virus/crimean-congo-hemorrhagic-fever-virus-strain-kosovo-hoti; Ref-SKU: 007 V-02504) [37, 51]. Virus titers were determined on SW-13 cells under the CO_2_^+^ culture condition using the TCID50 method (see below). Vero E6 cells were purchased from the American Type Culture Collection (ATCC, CRL-1586). SW-13 cells (ATCC, CCL-105) were a gift from Public Health Agency of Canada [52, 53]. Passage 32 was used for both cell types in comparative experiments.

Vero E6 cells were cultured in DMEM (Wisent, 319-005-CL), which contains L-glutamine (584 mg/L), sodium pyruvate (110 mg/L) and glucose (4.5 g/L), supplemented with 10% fetal bovine serum (FBS) (ThermoFisher Scientific, 12483020) and 1 × antibiotics (100 IU/ml penicillin and 100 µg/ml streptomycin) based on dilution of a 100 × penicillin streptomycin solution (Wisent, 450-201-EL). The culture was supplied with 5% CO_2_. Cells were split at a ratio range of 1:4 to 1:10 for subcultivation after reaching confluence.

SW-13 cells were tested for two different culture conditions: without CO_2_ versus with CO_2_. These culture types were named SW-13 CO_2_^−^ and SW-13 CO_2_^+^, respectively. SW-13 CO_2_^−^ cells were seeded in Leibovitz’s L-15 medium (ThermoFisher Scientific, 11415064), supplemented with the same 10% FBS and 1 × antibiotics as above. Growth was maintained at 37 °C in the absence of CO_2_ supply. Cells were split at a ratio range of 1:3 to 1:8 for subcultivation after reaching confluence. SW-13 CO_2_^+^ cells were cultured under the same condition as that for Vero E6, based on DMEM media and the presence of 5% CO_2_. Cells were split at a ratio range of 1:3 to 1:10 for subcultivation after reaching confluence.

### Imaging

Cell cultures were observed for morphological features including CPE under a CKX41 Inverted Microscope using the Olympus cellSens Standard software (Olympus Life Science). In plaque assays (see below), plaques were imaged under an EVOS M7000 Imaging System using the EVOS M7000 software (ThermoFisher Scientific).

### CCHFV infection of cell cultures

In 6-well culture plates, Vero E6, SW-13 CO_2_^−^ and SW-13 CO_2_^+^ cells reaching approximately 90% confluence (above 80% and below 100%) were inoculated with CCHFV at a multiplicity of infection (MOI) of 0.0001, based on the viral titer quantified using SW-13 CO_2_^+^ cells. The same volume of aliquots from the same viral stock was used for inoculations in order for all the cell cultures to physically receive the same amount of viral particles. CCHFV viral stocks were diluted in DMEM (without serum or antibiotic), for Vero E6 and SW-13 CO_2_^+^ cells or in Leibovitz’s L-15 medium (without serum or antibiotic), for SW-13 CO_2_^−^. After removal of old culture media, 1 ml of the viral dilution were added onto the cells. Viral adsorption was allowed for one hour at 37 °C with or without CO_2_ depending on cell culture types, and with gentle rocking on a rocker. 5 ml of maintenance media (DMEM + 2% FBS + 1 × antibiotics) were then added on top of the inoculum. The resulting viral cultures were maintained for 7 days. On a daily basis, CPE was monitored and samples of the culture supernatants were taken and stored at -80 °C for viral load and titer analysis at a later time. The morphological changes in CCHFV-infected cell cultures were noted descriptively and presented in microscopic images.

### Viral load analysis

Culture supernatant samples were inactivated by the TriPure Isolation Reagent (Sigma-Aldrich, 11667165001) by mixing 100 μl supernatants with 900 μl TriPure in a 1.5 ml Eppendorf tube. Purification of CCHFV RNA was then performed using the MagMAX CORE Nucleic Acid Purification Kit (ThermoFisher Scientific, A32700) on a KingFisher magnetic particle processor (ThermoFisher Scientific, A31508). Viral RNA concentrations were quantified using an RT-qPCR method targeting the S segment of the CCHFV genome previously published by Koehler et al. [54] with slight modifications. Specifically, the primer pair and probe were custom-synthesized by Integrated DNA Technologies, with the following names and sequences. CCHF-SF2: GGAVTGGTGVAGGGARTTTG, CCHF-SR2: CADGGTGGRTTGAARGC, and CCHF-N2: 6-FAM/CAARGGCAA/ZEN/RTACATMAT/IABkFQ. The following were diluted in H_2_O in a 20 µl reaction: 5 µl of 4 × TaqMan Fast Virus 1-step master mix (ThermoFisher Scientific, 4444432), 0.8 µl of 10 µM CCHF-SF2, 0.8 µl of 10 µM CCHF-SR2, 0.4 µl of 10 µM CCHF-N2, and 5 µl of template RNA. In vitro-transcribed S-segment RNA of CCHFV IbAr10200 [8] was serially diluted and used as standards for quantification of viral RNA copy numbers. Reactions were run and quantified on an Applied Biosystems 7500 Real-Time PCR System (ThermoFisher Scientific).

### CCHFV plaque assays

A carboxymethylcellulose (CMC) immobilizing overlay was used to restrict viral replication to the sites of initial infection, leading to the formation of plaques, foci of cell degradation or foci of viral accumulation in cells that are not degraded. For plaque visualization, a crystal violet staining of the cellular monolayer was tested to contrast-reveal potential empty spots resulting from cellular degradation (in Vero E6, SW-13 CO_2_^−^ and SW-13 CO_2_^+^ cells), and an immunostaining was tested to detect potential spots of viral antigen accumulation (in SW-13 CO2 + cells).

To prepare the overlay media (e.g. 300 ml of 1.75% CMC media), 5.25 g of CMC (Sigma-Aldrich, C4888) was dissolved in 223.7 ml of H2O with heating and stirring. The solution was then autoclaved at 121 °C for 15 min and cooled down to room temperature. Finally, the below listed were added, followed by mixing: 30 ml of 10 × DMEM (Wisent, 319-905-EL), 12 ml of 7.5% BSA Fraction V (Wisent, 809-095-EL, 14.8 ml of 7.5% NaHCO3 (Wisent, 609-105-EL), 7.5 ml of 1 M HEPES (Wisent, 330-050-EL), and 3 ml of each of the following: 0.4 g/L Folic Acid (Wisent, 609-315-QL), 200 mM GlutaMAX Supplement (ThermoFisher Scientific, 35050061), 100 mM Sodium Pyruvate (Wisent, 600-110-EL) and 100 × Penicillin Streptomycin (Wisent, 450-201-EL). The media were stored at 4 °C and upon use were warmed to 37 °C.

On the day of plaque assay start, or zero day post-infection (0 DPI), cells cultured in 48-well plates reaching confluence were inoculated with virus serially tenfold diluted in serum-free media (DMEM for Vero E6 or SW-13 CO_2_^+^ cells, or Leibovitz’s L-15 for SW-13 CO2- cells) at 100 µl/well, following the removal of old culture media. The inoculated viral dilutions were calculated/estimated to include a condition for the formation of around 100 plaques/well, to facilitate plaque visualization and counting. After viral adsorption for 1 h with rocking as described above, 0.5 ml of 1.75% CMC media were added and the plates were incubated without rocking to allow plaque development. Durations of the incubation tested in the crystal violet staining experiments ranged from 3 to 11 DPI. In the immunostaining experiments, 3, 4, 5 and 12 DPI were tested. The plates were then fixed overnight by filling up each well with 10% formalin (Fisher Scientific, SF100-4). Following fixation, the formalin-CMC media were discarded into a sealable container, where they were mixed with Formalex Green (Sasco Chemical Group) at a 4:1 ratio and left overnight before disposal. Residual formalin-CMC media on the plates were removed by gently and thoroughly rinsing the plates under tap water. The plates were angled so that the water did not directly land on the fixed cell monolayer to avoid excessive impact. Following the rinse, the plates were crystal-violet stained or immunostained as below to detect plaque formation.

For crystal violet staining, 100 µl of 0.5% crystal violet (in 80% methanol) was added to each well, followed by incubation at room temperature for 30 min. After removal of crystal violet and rinse with water, the plates were examined for plaques. To make 0.5% crystal violet, 0.5 g crystal violet (Sigma-Aldrich, C6158) was dissolved with 20 ml H2O and 80 ml methanol.

For immunostaining, the plates were subject to two extra washes with purified H2O (500 µl/well) after the tap water rinse. 100 µl of 0.3% Triton X-100, diluted from a 10% concentrate (ThermoFisher Scientific, 85111), was then added, followed by incubation at room temperature for 10 min and two washes with 500 μl of 1 × TBS-T, which was diluted from a 10 × concentrate (ThermoFisher Scientific, 28360). An anti-nucleoprotein Crimean-Congo hemorrhagic fever virus antibody (Abcam, ab190657) was diluted at 1:1000 or 1:2000 in 1 × TBS-T and used at 100 μl/well, with incubation at room temperature for 1 h. After two washes with TBS-T, this was followed by the addition of 100 μl of Polymer-HRP anti-rabbit, part of the Dako EnVision +/HRP immunohistochemistry visualization kit (Agilent, K400311-2), and incubation at room temperature for 30 min. Following two washes with TBS-T and two washes with H_2_O, 100 μl of Dako EnVision+ substrate (Agilent, K346811-2) was added, which was freshly prepared based on 1 drop of DAB+ Chromogen per 1 ml of DAB+ Substrate Buffer. The plates were incubated at room temperature for 5 min, washed twice with H_2_O and allowed to dry. Plaques, in the form of a brown-color staining, were imaged under the microscope.

### TCID50 assay

The assay was based on the Spearman & Kärber algorithm, implemented by Marco Binder as a TCID50 calculator in an excel spreadsheet format [55]. The calculator can be downloaded from the following website (last accessed on 2022-11-30):https://www.researchgate.net/file.PostFileLoader.html?id=58dad730f7b67ea37125593f&assetKey=AS%3A476999471898624%401490736944531

We adapted the TCID50 calculator to CCHFV titration with slight modifications (Additional file 1). Based on the 96-well plate format, the circumferential wells (corner and edge wells) were not finally used for viral titration calculations, since SW-13 cells demonstrated susceptibility to an edge effect (described in Fig. 4A). However, those wells were still seeded and maintained throughout the titration process, as intended to shield the internal wells against effect of evaporation and thermal changes.

Two days prior to viral inoculation (-2 DPI), 48,640 SW-13 cells in 150 µl complete media (DMEM + 10% FBS + 1 × Pen Strep, for CO_2_^+^ culture) were seeded per well on a 96-well plate (viral titration plate). On inoculation day (0 DPI), cell confluence was expected to be at 80–90% but not higher than 100%. To prepare viral dilutions, 33 µl virus was mixed with 297 µl DMEM per well on a 96-well plate (viral dilution plate). A series of 1:10 dilutions were made following the same formulation, which could range from 1:10 to 1:10^10^. However, depending on the titer expectation, users could choose to test a narrower viral dilution range. For example, a 1:10 to 1:10^5^ range should roughly cover the need for titrating an un-concentrated viral stock with a moderately high titer. Therefore, a single 96-well viral titration plate could accommodate the titrations of two original viral stock samples. Alternatively, a dilution range could start at a higher dilution factor, such as 1:10^2^ or 1:10^3^. Subsequently, each of the viral dilutions was assigned to a column on the titration plate and involved six replicate wells. Due to the edge effect mentioned above, columns 1 and 12 and rows A and H were excluded. Following the removal of old media, 45 µl of viral dilution was added. The plate was then placed back into the cell culture incubator (37 °C, 5% CO_2_), on a rocker with a gentle rocking speed, to allow viral adsorption for one hour (1 h). Finally, 155 µl of maintenance media (DMEM + 2% FBS + 1 × antibiotics) were added on top of the 45 µl inoculum, and the titration plate was placed back to incubation (37 °C, 5% CO_2_). On 5 DPI, wells were examined for CPE. To calculate the viral titer, the following parameters were filled into the excel TCID50 calculator, where a TCID50/ml value was then generated automatically: volume per well = 45 µl, replicate wells per dilution = 6, dilution factor = 1:10, dilution in the first well = 1:10 (or as customized alternatively), and number of positive wells (CPE +) for each dilution condition as observed on the viral titration plate.

### Statistical analysis

Significance of difference in viral loads and titers from CCHFV cultures was determined by *Student’s t* test in GraphPad Prism. A p value less than 0.05 was defined as significant.

### Biosafety approval statement

All experiments involving infectious CCHFV were conducted in the containment level 4 (CL4) laboratory at the National Centre for Foreign Animal Diseases, Canadian Food Inspection Agency, following the institutional standard operating procedures.

## Results and discussion

### Identification of major cell lines used for CCHFV culture and titration in the literature

**Fig. 1:**
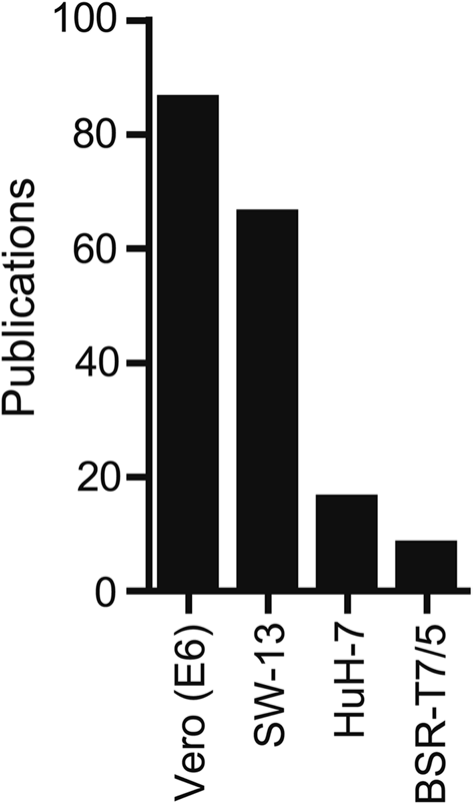
Major cell lines for CCHFV culture. Data are based on a PubMed search covering all-time literature. The search method is detailed in Materials and Methods. Graph shows number of publications in PubMed using each indicated cell line for CCHFV propagation and titration. Details of these publications, including PMID, publication year and article title, are provided in Additional file 2

We performed a comprehensive search for research papers describing CCHFV cell culture, which covered all-time literature in the PubMed and identified four cell lines of considerable popularity, Vero (E6) [4, 13, 14, 16–27], SW-13 [8, 17–21, 24, 26, 28–41], HuH-7 [17, 20, 21, 31, 33, 39, 56, 57] and BSR-T7/5 [4, 57–59] (Fig. 1 and Additional file 2).

The two most cited cell lines, Vero (E6) and SW-13 are both commercially available from ATCC, the leading global supplier of authenticated cells lines. Vero cells (epithelial-like) originated from the kidney tissue of a normal, adult African green monkey (*Cercopithecus aethiops*). Both the parental Vero cell line (ATCC, CCL-81) and a clone derived from the parental cell line, Vero E6 (ATCC, CRL-1586), have been used in CCHFV culture. However, Vero E6 constitutes the majority of cases as specified in the publications. Moreover, Vero E6 is sometimes referred to as “Vero” by users for simplicity [44], and therefore the actual number of studies using Vero E6 may be higher. In this study we chose to focus on Vero E6 considering its majority role as well as a supposed similarity among Vero cells. SW-13 (epithelial-like) was isolated from the adrenal gland and cortex of a human carcinoma patient (ATCC, CCL-105).

The other two cell lines, HuH-7 and BSR-T7/5, are not supplied by ATCC. HuH-7 was reportedly available from Apath, LCC [18]. It is also listed in the catalogues of the Japanese Collection of Research Bioresources (JCRB0403) and Fisher Scientific (NC0848302). The cell line was established from a human hepatocellular carcinoma [60]. BSR-T7/5 is not commercially available and for CCHFV culture has largely been used by researchers from the Centers for Disease Control and Prevention predominantly to rescue recombinant CCHFV [18, 57, 58]. This cell line is a derivative of the golden hamster (*Mesocricetus auratus*) cell line BHK-21 (kidney fibroblast; ATCC, CCL-10) and contains a stable integration of T7 RNA polymerase expression [61].

Following a comprehensive search, we found that there was a lack of description on the characteristics of CCHFV culture systems and on the technical details in propagation and titration methods in CCHFV literature. To address this gap, we carried out a detailed characterization and optimization in both Vero E6 and SW-13 cells. We focused on these two cell types based on the following considerations. Since Vero E6 and SW-13 have been used more extensively than other cell lines, we inferred that they may represent a superior combination of characteristics important in viral propagation and titration methods for broad accessibility and usability, such as commercial availability, high titer production, user-friendliness and reproducibility. Apart from the availability through the standard supplier ATCC, Vero E6 and SW-13 cells have proved easy to maintain with stable and consistent morphology, viability and performance in our previous cell culture experiments (SW-13 for CCHFV and Vero E6 for other viruses) [8, 55, 62–64]. Some authors consider SW-13 to be one of the most permissive cell lines for CCHFV with data depicting high titers generated in these cells comparable to those generated in HuH-7 and BSR-T7/5, the two relatively “newer” cell lines to the field [15, 33, 65]. Finally, SW-13 appeared to be the only cell line demonstrating a clear CCHFV-induced CPE among these and many other cell lines [15, 66–68]. This represents a unique advantage for quick and easy identification of CCHFV infection in cell cultures, particularly useful in experiments such as viral isolation and staining-free TCID50-based titration.

### Growth characteristics of host cell lines under different cultivation conditions

We first conducted comparative assessment on the growth characteristics of Vero E6 and SW-13 in the context of different culture conditions. We considered that factors related to the differential CO_2_ requirement in these cultures could be a major influential variable. Most mammalian cell lines are maintained in the presence of CO_2_. In a compatible medium, CO_2_ contributes to a stable physiological pH through the carbonate-bicarbonate buffer system. This was also applied to Vero E6 cells. ATCC recommends a culture condition with 5% CO_2_ in combination with a base medium, Eagle's Minimum Essential Medium (MEM) [69]. However, for CCHFV propagation, a modified version of this medium, Dulbecco's Modified Eagle Medium (DMEM), is popularly used for Vero E6 cells instead, which is also CO_2_ compatible [4, 13, 14, 16]. Containing higher concentrations of nutrient supplements [70], DMEM is widely used for growing different types of mammalian cells [71], and “is the most broadly suitable medium for many adherent cell phenotypes among defined media for cell and tissue culture” with enhancement in nutrients such as amino acids [72, 73]. Therefore, for consistency with other researchers, we chose to culture Vero E6 cells in DMEM-based media along with 5% CO2. As for SW-13, in contrast, the standard culture condition is without CO_2_ according to ATCC [74] and the initial cell line report [75]. The special base medium, Leibovitz's L-15, was devised for use in a free gas exchange with atmospheric air, whereas a CO_2_ and air mixture is detrimental to cells. This culture condition (SW-13 CO_2_^−^) was also previously used to culture CCHFV [28, 32, 38, 49, 50, 53]. In this study we hypothesized that a CO_2_ compatible culture condition could make a difference in the performance of SW-13. To test this, we applied the Vero E6 culture condition, based on DMEM and CO_2_ supply, to SW-13 (SW-13 CO_2_^+^). Cell growth characteristics were then observed and compared under the three culture conditions: SW-13 CO_2_^−^, SW-13 CO_2_^+^ and Vero E6 (CO_2_^+^). The comparative assessment of the culture conditions was conducted simultaneously in side-by-side incubators without CO2 versus with CO2.

**Fig. 2:**
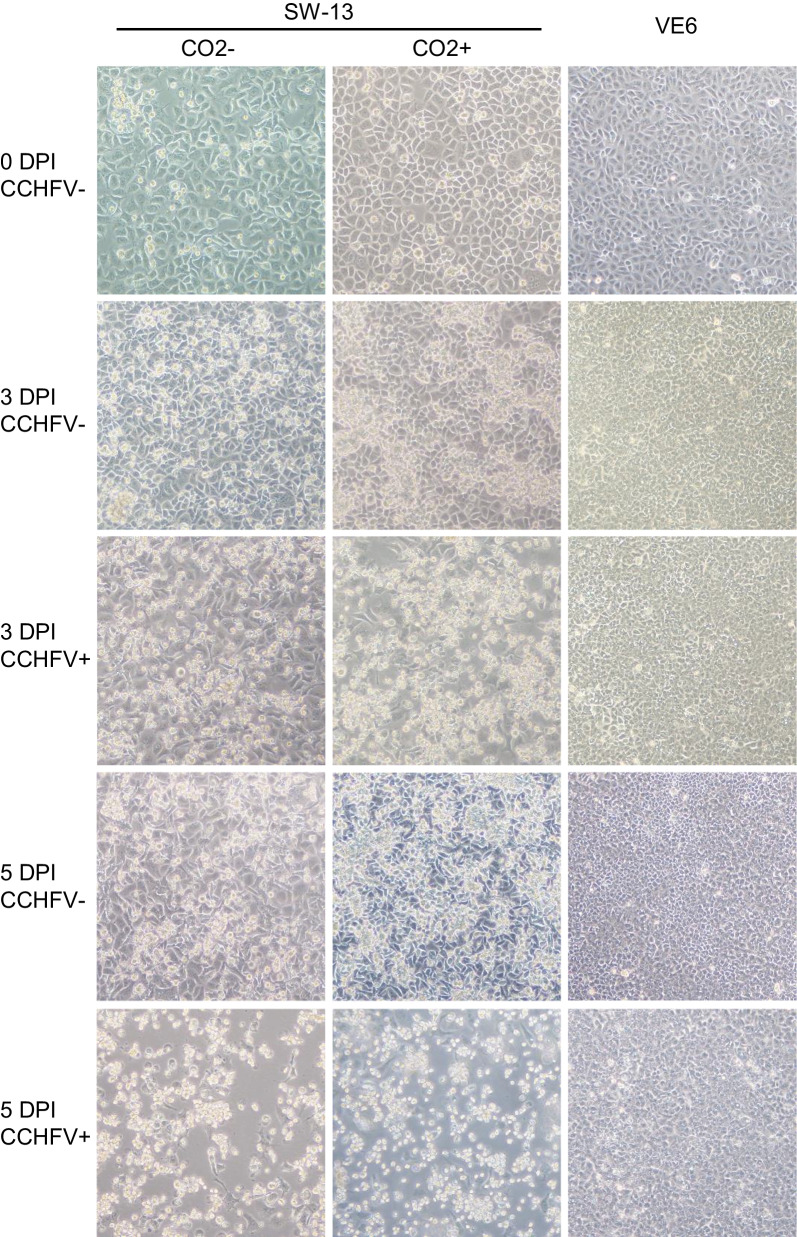
Growth characteristics of host cell lines. SW-13 cells cultured in the absence or presence of CO2 (CO2- or CO2 +) and Vero E6 (VE6) cells were infected with CCHFV (CCHFV+) or mock-infected (CCHFV−) and imaged at the indicated time points. DPI, days post-infection. Images represent seven experiments (n = 7) showing similar patterns (each experiment based on two replicates)

As adherent cells, SW-13 and Vero E6 were both found to form a monolayer attached to the culture vessel. The growth of Vero E6 cells was restricted within the monolayer (contact-inhibited), even after reaching confluence (Fig. 2). In contrast, SW-13 cells (CO_2_^−^ and CO_2_^+^) were not contact-inhibited and grew over a confluent monolayer forming clusters of cells during extended culture (Fig. 2). These clusters were part of a healthy, actively growing culture, and their viability was confirmed by trypan blue exclusion test. A small number of singlet cells, largely dead cells (trypan blue staining positive), were occasionally seen. SW-13 CO_2_^−^ cells had a relatively higher presence of these singlet cells than CO_2_^+^ cells, indicating that the CO_2_^+^ condition has a positive effect on cellular viability. In addition, the CO_2_^+^ condition was also found to enhance adherence. SW-13 CO_2_^+^ cells attached to the culture vessel more tightly than CO_2_^−^ cells, although less so than Vero E6 cells. This was noted by the difference in the trypsin digestion time to detach the monolayer, and the ranking among the three cell culture types in this aspect was: Vero E6 > SW-13 CO_2_^+^  > SW13 CO_2_^−^. Consistently, following formalin fixation during plaque titration assays (described below), SW-13 CO_2_^−^ cell layers were easily detached whereas SW-13 CO_2_^+^ and Vero E6 cell layers remained stable. It is likely that the CO_2_^+^ culture condition increases the amount of extracellular matrix produced by SW-13 cells or/and promotes the formation of stronger adhesion structures.

The most dramatic effect of the CO_2_^+^ culture condition was on the growth rate. The SW-13 CO_2_^−^ cells grew substantially slower than Vero E6 cells (with estimated doubling times: 1.5 days versus 1 day). These are consistent with previously reported data [76–78]. The SW-13 CO_2_^+^ cells, however, grew significantly faster than SW-13 CO_2_^−^ cells, at a rate close to that of Vero E6 cells. Altogether, these observations indicate that the CO_2_^+^ culture condition leads to more viable, stronger and more active SW-13 growth, with potential impact on viral production expected.

### Replication characteristics of CCHFV in host cell lines and an optimal viral propagation method

We inoculated the CCHFV IbAr10200 strain in SW-13 CO_2_^−^, SW-13 CO_2_^+^ and Vero E6 cultures (MOI 0.0001) and daily for seven days monitored morphological changes and collected supernatant samples for analysis of viral loads (RNA) and titers (infectious virus). The use of a low MOI was intended to minimize a potential inhibitory effect by interferon carryover on the initiation and establishment of infection. Interferon responses are a known antiviral mechanism against CCHFV infection [15, 47, 52, 79–82] and these responses have been reported in Vero and SW-13 cells [15]. Pre-treatment (24 h or 2 h before the infection) or early treatment (1 h post-infection) but not delayed treatment (6 h post-infection) of cell cultures with interferon was previously found to result in reduced viral titers, suggesting that the early stage of CCHFV infection in the cell culture context is more sensitive to interferon responses and the sensitivity drops once the virus is replicating [79]. Therefore, to facilitate the start of viral replication, we used the low MOI to limit the introduction of interferons or other inhibitory contaminants likely present in CCHFV viral stocks produced from cell cultures. Furthermore, the low MOI (with low input of viral inoculation) did not appear to result in a reduction in viral productivity compared to a higher MOI of 0.001 (Additional file 3).

The Vero E6 culture largely remained as an intact monolayer throughout the process, in the absence or presence of CCHFV infection (Fig. 2). There was no significant accumulation of cells in the suspension phase of the culture. Over time, individual cells in the monolayer appeared to be compressed in size and the monolayer became more compact. However, overall there was no significant CPE observed (Fig. 2). In both SW-13 CO_2_^−^ and CO_2_^+^ cultures, extended growth led to accumulation of cell clusters on top of a confluent monolayer, as observed in control cells throughout the process and in CCHFV-infected cells prior to any CPE appearance. The cluster accumulation was to a higher extent in the CO_2_^+^ cultures, possibly due to faster growth (Fig. 2). In sharp contrast to Vero E6, both SW-13 CO_2_^−^ and CO_2_^+^ demonstrated a clear and dramatic CPE pattern, starting at three days post-infection (3 DPI). It included several typical morphological changes (Fig. 2). The monolayer was disrupted, with cells detached leaving empty areas on the growth surface of culture vessel. Cells remaining attached deformed into an elongated shape. The clustered structure originally found in the suspension cell population was also subjected to disruption. There was an accumulation of singlet cells in suspension with a rounded shape, smaller sizes and an uneven-looking membrane texture (Fig. 2). At a late stage of CPE (5 DPI and later), the entire culture turned into individual pieces of cellular debris (Fig. 2). Accompanying these changes, CPE was also found to affect the culture pH, which was tracked by the color of phenol red, a pH indicator dye contained in the SW-13 media. In control cultures, extended growth led to a change in medium color from red to orange/yellow, demonstrating a decrease in pH, presumably due to the accumulation of metabolites. In CCHFV-infected cultures, however, CPE was associated with the medium color remaining in red, indicating an inhibition of cellular metabolism. Thus, medium color could serve as a quick and easy accessory indicator of the presence or absence of CPE, along with the primary morphological features of CPE as described above.

**Fig. 3:**
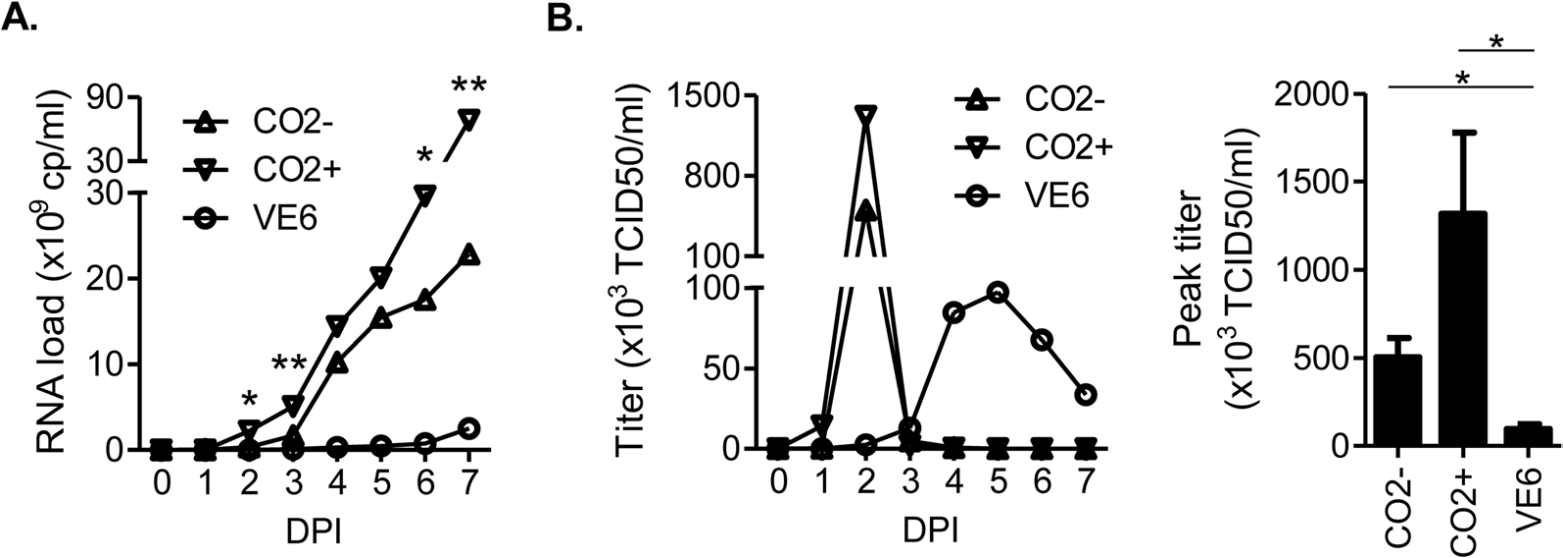
Replication kinetics of CCHFV in host cell lines. SW-13 CO2-, SW-13 CO2+ and Vero E6 (VE6) cells were infected with CCHFV, and the culture supernatants were analyzed for viral loads and titers, based on seven independent experiments. There were two replicates per experiment, the mean of which was used as the value representing each experiment (experiment value). Line graphs depict the mean of experiment values from all independent experiments. Bar graph indicates the mean ± SEM of experiment values from all independent experiments. **A** Viral loads (RNA concentrations) at indicated time points. cp, copies. DPI, days post-infection. Asterisks indicate statistically significant difference found between SW-13 CO2- and SW-13 CO2 + at the corresponding time point: *p < 0.05 and **p < 0.01. **B** Viral titers (infectious virus concentrations). TCID50, Median Tissue Culture Infectious Dose. All viral titers were determined on SW-13 CO2 + cells using the TCID50 method. Left panel shows viral titers at indicated time points. Right panel compares the peak viral titers. Both the SW-13 CO2− and SW-13 CO2+ cultures demonstrated significantly higher peak viral titers than the VE6 culture: *p < 0.05. The CO2+ culture shows trend of a higher peak viral titer than CO2− (*p* = 0.14)

The viral loads (viral RNA level as indicated by S-segment RT-qPCR) in all the three culture conditions maintained a daily increase through the time course, reaching the highest level at the end (7 DPI). SW-13 CO_2_^+^ cells produced significantly higher levels of viral loads than CO_2_^−^ cells, while both the SW-13 culture conditions were by far more productive than Vero E6 (Fig. 3A). The peak viral loads in SW-13 CO_2_^−^, SW-13 CO_2_^+^ and Vero E6 cells were 2.28 × 10^10^, 6.83 × 10^10^ and 2.50 × 10^9^ RNA copies/ml, respectively (Fig. 3A). Statistical significance of difference was confirmed at several time points between the SW-13 CO_2_^−^ and SW-13 CO_2_^+^ conditions as indicated in Fig. 3A. These data suggested the most active CCHFV replication in SW-13 CO_2_^+^ cells. The viral titers, however, did not follow the continuously-increasing pattern seen with the viral loads, and demonstrated distinct, cell type-dependent kinetics (Fig. 3B). In SW-13 CO_2_^−^ and CO_2_^+^ cells, the titers peaked quickly, at 2 DPI, and then dropped sharply. In Vero E6 cells, the titer increased in a slower manner and reached a peak level around 5 DPI, followed by decrease at a moderate rate (Fig. 3B). The peak titers in SW-13 CO_2_^−^ (5.03 × 10^5^ TCID50/ml) and CO_2_^+^ cells (1.32 × 10^6^ TCID50/ml) were both significantly higher than that in Vero E6 cells (9.72 × 10^4^ TCID50/ml). SW-13 CO_2_^+^ cells demonstrated a trend of producing a higher peak titer than CO_2_^−^ cells, although the difference did not reach statistical significance (p = 0.14) (Fig. 3B). These data suggested to us that SW-13 CO_2_^+^ could be chosen as an optimal culture system for high-titer and rapid CCHFV propagation. This conclusion, however, was drawn in the context of viral harvest directly from the culture supernatants.

We discuss here the possibility that titers obtained on viruses released into the supernatants may not necessarily correlate well with their counterparts achievable by isolating intracellular viruses. Indeed, Vero E6 cells were lysed to prepare CCHFV stocks in some previous studies [13, 14, 47], presumably due to higher abundance of intracellular viruses relative to that of secreted viruses. Nevertheless, we prefer direct viral collection from supernatants over isolation from cell lysis, in forming the basis for a standard, optimal CCHFV propagation method. Compared to cell lysis, which introduces extra experimental steps in a high containment context, supernatant harvest is simple and user-friendly, with minimal technical complexity. Furthermore, cell lysis is expected to release into the isolation buffer a range of intracellular contents, including not only mature, functional CCHFV virions, but also intermediate products along the viral replication pathway such as varieties of viral RNA transcripts, proteins and large molecular complexes, as well as host cell-derived intracellular contaminants. These could impose complicated biological effects on downstream applications using the resulting viral stocks.

In contrast, in SW-13 CO_2_ + cells, the highest CCHFV titer can be harvested on 2 DPI (Fig. 3B), before the start of CPE (3 DPI) (Fig. 2). In this context, it is expected that host cells maintain integrity, mature virions exit host cells through the budding mechanism along the natural viral replication pathway, and no other intracellular substances are released into the supernatants. Therefore, the viral harvest should be composed of a clean, homologous population of mature CCHFV virions, with minimal intracellular contaminants. This could also be part of a conceivable scenario underlying the discrepancy between the kinetic profiles of viral loads (RNAs) and titers (infectious viruses) in SW-13 cultures (Fig. 3). At pre-CPE time points, heathy host cells actively produced and released viable virions, leading to the accumulation of infectious titers in the supernatants, where viral RNAs were mostly limited to viral genomes packaged in intact virions. Following the start of CPE, the productivity of host cells in supplying new virions was inhibited, and the existing virions seemed to be inactivated, presumably due to exposure to changes in the media in the presence of contaminants leaked from dying host cells. Together these resulted in a quick loss of functional, infective viruses. In contrast, viral RNAs were continuously accumulated, with more and more cells degraded over time releasing intracellular contents containing them.

### Plaque assays for CCHFV titration

**Fig. 4:**
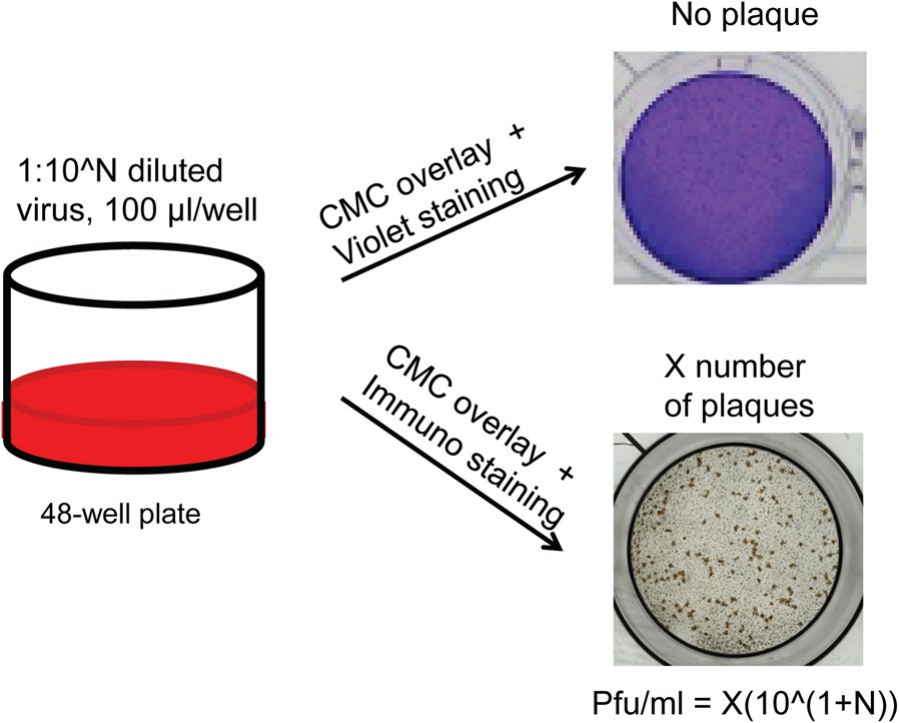
Plaque assays for CCHFV titration. Cell cultures in 48-well plates were infected with CCHFV as indicated. A carboxymethylcellulose (CMC) immobilizing overlay was added to restrict viral replication to the sites of initial infection, for the formation of plaques, foci of cell degradation (empty spots in monolayer), or foci of viral accumulation in cells that are not degraded (monolayer intact). The detection of these two possible types of plaques was tested using crystal violet staining, which would stain cellular monolayer into a violet color background, contrast-exposing the cellular degradation-based plaques as colorless empty spots, and using immunostaining to show the plaques based on intracellular viral antigen accumulation, respectively. Crystal violet staining failed to identify any plaques in CCHFV-infected SW-13 CO2−, SW-13 CO2+ or Vero E6 cells at any daily time point between 3 and 11 DPI. Immunostaining successfully revealed plaques in CCHFV-infected SW-13 CO2 + cells, at all the four tested time points, 3, 4, 5 and 12 DPIs, while the other cell culture types were not tested. A representative image was shown for each of the staining methods

We next sought to apply the characteristics of CCHFV propagation in host cell lines to the detection and quantification of infective viruses. We reasoned that, due to CPE in SW-13 cells, infectious units of CCHFV would create individual empty spots in the monolayer where host cells were detached, which could be contrast-highlighted by color staining of neighboring cells that remain attached. This was then tested in titration experiments comparing SW-13 CO_2_^−^, SW-13 CO_2_^+^ and Vero E6. Following viral inoculum adsorption on 48-well cell culture plates, a semisolid CMC overlay was imposed to restrict the diffusive spread of viruses derived from any individual infectious unit. The titration plates were maintained for daily time points ranging from 3 to 11 DPI, followed by formalin fixation. Cells were finally stained with a crystal violet dye to reveal potential plaques, spots with viral replication from single infectious units. As expected, no plaques were found with Vero E6, which was consistent with the absence of CPE. Unexpectedly, however, this was also the case with SW-13 CO_2_^−^ and SW-13 CO_2_^+^ (Fig. 4).

We speculated that the CMC overlay might have inhibited the degradation of CCHFV-infected SW-13 cell monolayers. An inhibitory effect of CMC on CCHFV replication could also cause failure of plaque formation, however this is much less likely since the CMC condition had not been previously found to have such effect in plaque assays of a variety of viruses [62, 83]. In turn, an immuno-plaque assay was attempted. Titration plates with CCHFV-infected SW-13 CO_2_^+^ cells under CMC overlays were formalin-fixed at 3, 4, 5 and 12 DPI, cells were permeabilized and CCHFV detection was performed using a commercially available antibody against the viral nucleoprotein coupled with horseradish peroxidase (HRP)-Diaminobenzidine (DAB) staining. CCHFV plaques were successfully exhibited through this staining protocol (Fig. 4). These data suggest that the CMC overlay did not have any notable inhibitory effect on CCHFV replication but likely impeded cellular degradation. As a result, non-degraded or partially degraded cells on the spots of CCHFV replication were then fixed by formalin. These spots were not distinguishable from surrounding cells by crystal violet staining since they were not empty and their cellular contents would stain positive by the dye. However, the viral antigens on these spots were successfully detectable by immunostaining with a CCHFV-specific antibody.

The immuno-plaque assay was developed using only SW-13 CO_2_^+^ cells, but not SW-13 CO_2_^−^ or Vero E6 cells. The main reason was that it would be logistically the easiest to use the same one culture type throughout both viral propagation and titration experiments, and SW-13 CO_2_^+^ had been identified as optimal for CCHFV propagation. Moreover, the monolayer of SW-13 CO_2_^−^ cells tended to fall off as found in the crystal violet staining experiments, possibly due to less tight adhesion than that of SW-13 CO_2_^+^ or Vero E6 cells.

Notably, the use of a commercially available CCHFV antibody rather than an in-house produced antibody is anticipated to enhance the reproducibility and standardization of the immuno-plaque assay across different laboratories. In addition, based on the colorimetric readout, CCHFV positive culture wells with plaque staining could be quickly identified with naked eyes and individual plaques could be counted under a light microscope of low magnification capacity or even with a simple glass magnifier. Therefore, the colorimetric readout, compared to the fluorometric readout, which requires fluorescent microscopy, adds to the suitability of immunostaining for the detection of CCHFV in resource-limited setting.

### A TCID50 assay for CCHFV titration

**Fig. 5:**
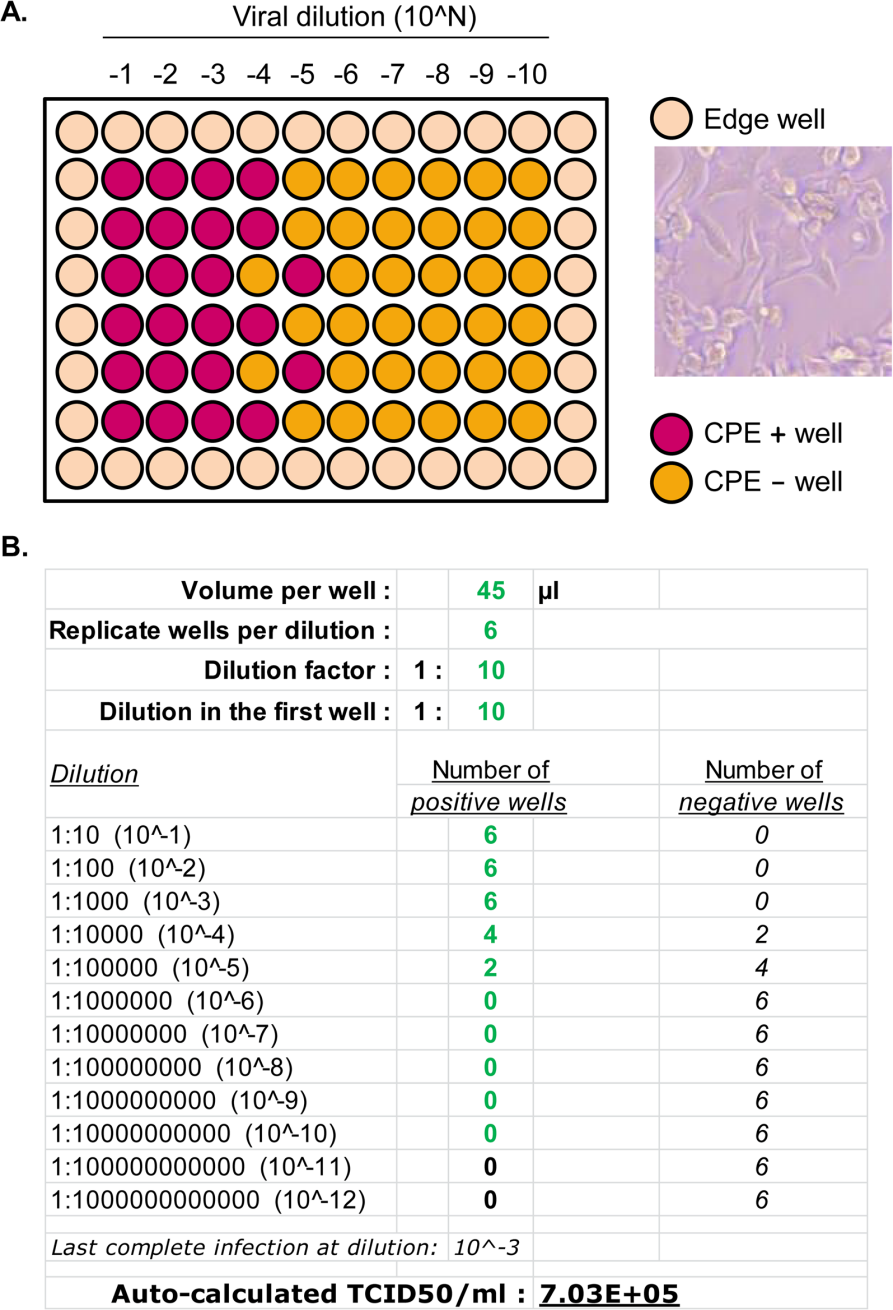
The TCID50 assay. **A** Schematic of a 96-well viral titration plate. An original CCHFV sample was serially diluted as indicated and inoculated onto SW-13 CO2 + cells. The peripheral wells, represented with light pink color, were excluded from usage for the assay, due to an edge effect on cell morphology as illustrated in the microscopic image. Each dilution condition was assigned onto one column of wells, consisting of six replicates. CCHFV positive wells (CPE+, represented in red) or negative wells (CPE−, orange) were distinguished by the presence or absence of typical morphological changes characteristic of CPE, such as monolayer disruption and cellular deformation (detailed in text of Results and Discussion). Medium colors were a quick accessory indicator of CPE status, based on phenol red, a pH indicator. Extended culture, with accumulation of metabolites, led to a color change from red to orange (pH decrease) in CPE- wells, whereas the color remained red (sometimes slightly purplish) in CPE+ wells, where metabolism was inhibited. Colors used here, for illustration purpose, may not precisely reflect the real colors seen in experiments. **B** Highlight of the TCID50 calculator. This is in an excel spreadsheet format. Represented in green were customizable design parameters and numbers of positive (CPE+) wells such as those observed on the titration plate in A. Based on these inputs from the user, the viral titer in TCID50/ml was automatically calculated

Based on the prominent CPE in SW-13 cells, we tested an alternative way to quantify CCHFV titers. We adopted a TCID50 method which only depends on CPE patterns to calculate the number of infectious virus particles [55] (Fig. 5). In this method, a series of CCHFV dilutions were added to SW-13 CO_2_^+^ cells in a 96-well plate format. Each dilution was assigned to one column of wells representing replicates. At 5 DPI, cells were inspected for CPE and each well was classified as infection positive or negative. The overall infection patterns were then used to calculate the viral titer by the Spearman & Kärber algorithm. This classical mathematical approach has recently been implemented as an automatic titer calculator in an excel spread sheet format. We adapted the excel calculator to CCHFV titration, by making slight modifications based on culture parameters established in this study (Additional file 1). Following the input of the numbers of CCHFV positive wells, the titer in TCID50/ml is automatically calculated and displayed (Fig. 5). The TCID50 assay represents a cost-effective, simple and user-friendly CCHFV titration method by saving reagents and efforts related to antibody staining and by using automatic calculation.

### The propagation and titration methods can be applied to a more pathogenic CCHFV strain

IbAr10200 is the most common CCHFV strain used in laboratory research [40, 42, 43, 84, 85]. Representing CCHFVs in many aspects, it has been contributing to research findings that can be generalized [40]. Thus, IbAr10200 was adopted in this study as a representative CCHFV strain. However, current research towards CCHFV medical countermeasures is increasingly utilizing more pathogenic strains such as Kosovo Hoti (hereafter Hoti) [17, 20, 37, 51, 86]. Using Hoti we performed confirmatory test of our CCHFV propagation and titration methods based on SW-13 CO2 + cells (Additional file 3). Hoti was found to generate prominent CPE and clear plaque staining patterns similar to those generated by IbAr10200. Based on these, the TCID50 and plaque assays were successfully applied to the quantification of viral titers. Peak titers were achieved at the magnitudes of 10^6^–10^7^ pfu or TCID50 per ml. Compared to IbAr10200, Hoti demonstrated a relatively slower growth kinetics, with CPE appearing on 5 DPI and viral titer peaking on 3 DPI. These results indicate that the CCHFV propagation and titration methods can be applied to a more pathogenic CCHFV strain, not limited to IbAr10200.

## Conclusions

This study provides a detailed analysis on the growth characteristics of Vero E6 and SW-13, CCHFV host cell lines of top popularity and with standard commercial availability, under different culture conditions (concerning CO_2_ and base media), and subsequently on the replication kinetics of CCHFV in these cultures. The comparative evaluations identified SW-13 CO_2_^+^ as an optimal system for productive and fast CCHFV propagation. For viral titration, this optimal culture type was used to establish a plaque assay based on immunostaining with a commercially available CCHFV antibody and a colorimetric readout as well as a TCID50 assay based on a clear CPE pattern and a simple excel calculator. Additionally, the entire CCHFV propagation and titration process can be fulfilled using a single cell culture condition, which is also based on the same atmospheric requirements as other CL4 viruses, requiring no dedicated incubator without CO_2_ supply that would occupy additional CL4 lab space.

In conclusion, the findings from this study have laid a foundation for reproducible, standardized and user-friendly methods for CCHFV culture and titration that can be commonly shared across the CCHFV research community.

## Supplementary Information

**Additional file 1**. The TCID50 calculator as an excel spread sheet.

**Additional file 2**. List of research papers describing major cell lines for CCHFV culture and titration.

**Additional file 3**. Application of CCHFV propagation and titration methods to the Kosovo Hoti strain.

## Data Availability

All data generated or analyzed during this study are included in this published article and its supplementary information files.
