# Supplementary material for: Comparative characterization of Crimean-Congo hemorrhagic fever virus cell culture systems with application to propagation and titration methods
Source: Virol J. 2023 Jun 19;20:128. doi: 10.1186/s12985-023-02089-w (PMC10280830; doi:10.1186/s12985-023-02089-w)
Supplement: Supplementary file 3 — Additional file 3. Application of CCHFV propagation and titration methods to the Kosovo Hoti strain. [file 12985_2023_2089_MOESM3_ESM.pdf]

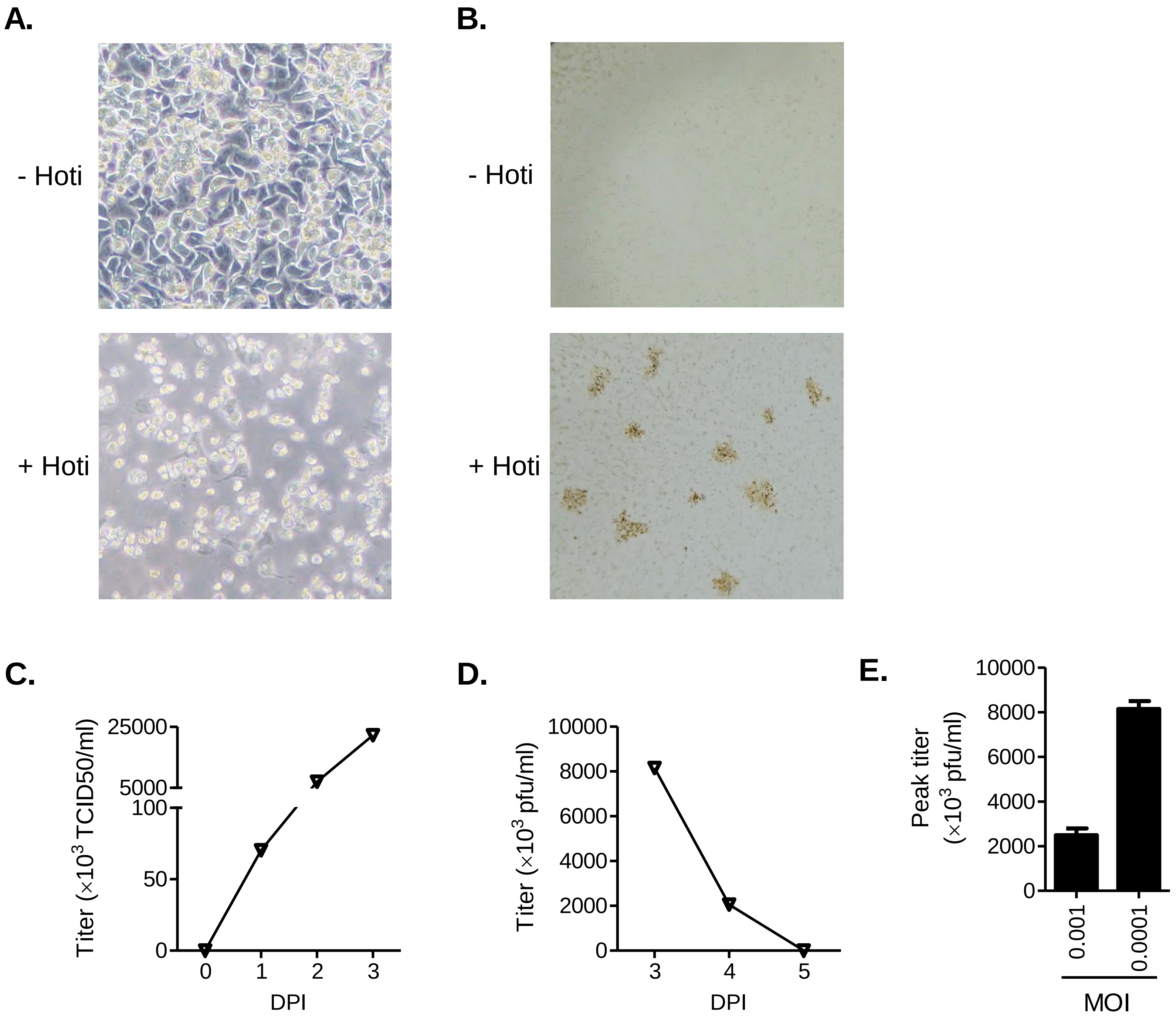

Additional file 3. Application of CCHFV propagation and titration methods to the Hoti strain. **A and B.** SW-13 CO_2^+ control cells (- Hoti) or cells inoculated with CCHFV Hoti (+ Hoti, MOI = 0.0001) were examined for morphological difference by microscopy in A or plaque formation by the immunostaining-based plaque assay in B. A prominent CPE appeared on 5 DPI in Hoti-infected cells. Plaques were detected by staining of 3-DPI cells. Images represent three experiments showing similar patterns. **C and D.** From cell cultures infected with Hoti as in A and B supernatants were collected at the indicated DPI time points and quantified for viral titers using the TCID50 assay in C or the plaque assay in D. Graphs represent mean of duplicates. 3 DPI was identified as the time point of peak viral titer for Hoti. **E.** SW-13 CO_2^+ cultures infected with Hoti at the indicated MOIs were compared for peak viral titers. Bars represent mean ± SEM of duplicates. Lowering MOI did not appear to cause reduced viral productivity.
